# Prevalence of periodontal disease among Indigenous and non-Indigenous populations: protocol for systematic review and meta-analysis

**DOI:** 10.1186/s13643-022-01913-8

**Published:** 2022-03-12

**Authors:** Sonia Nath, Brianna Poirier, Xiangqun Ju, Kostas Kapellas, Dandara Haag, Lisa Jamieson

**Affiliations:** Australian Research Centre for Population Oral Health, Adelaide Health & Medical Sciences Building, The University of Adelaide, Adelaide, SA 5005 Australia

**Keywords:** Indigenous, Native people, Oral health, Periodontitis, Prevalence

## Abstract

**Background:**

Indigenous populations globally experience worse oral health than their non-Indigenous counterpart. Globally, the occurrence of periodontal diseases such as gingivitis and chronic periodontitis is high among Indigenous people. This systematic review aims to quantify, at a global level, the prevalence of periodontal disease among Indigenous populations compared to non-Indigenous populations.

**Methods:**

This review will only consider studies that have reported the prevalence (%) of periodontal disease among Indigenous and compared against non-Indigenous populations. Studies that have no comparative population or data only on one particular population or lack of data on periodontal clinical assessment will be excluded. An electronic search will be conducted using keywords and appropriate MeSH terms across several databases capturing both published and unpublished articles. The search will be conducted from the time of database inception to February 2021. After the initial search, duplicates will be removed, and the remaining titles and abstracts will be assessed for eligibility. The full text of eligible studies will be assessed by two independent reviewers who will also complete the critical appraisals and data extraction. Outcomes measures would be the mean prevalence (%) and standard deviation of periodontal disease among Indigenous and non-Indigenous populations. From the selected studies, we will conduct a random-effects meta-analysis using standardized mean difference as the effect measure. Forest plots will be used for the visualization of differences in the prevalence of periodontitis. A subgroup analysis will be conducted based on the definition of periodontitis, age, publication type, and geographical location. Heterogeneity among studies will be assessed by *I*^2^ and chi-square test. Egger’s test and funnel plots will be used to assess publication bias.

**Discussion:**

Our systematic review and meta-analysis will facilitate an increased understanding of the magnitude of periodontal disease inequalities that exist globally for Indigenous populations through pooled prevalence estimates. The findings will be helpful to design selective targeted preventive and interventional strategies for periodontal disease for reducing oral health inequalities at a global level.

**Systematic review registration:**

PROSPERO CRD42020188531

**Supplementary Information:**

The online version contains supplementary material available at 10.1186/s13643-022-01913-8.

## Background

Across global geographic and cultural differences, Indigenous peoples experience similar difficulties in defending their sovereignty and are among the most disadvantaged populations [1, 2]. According to a United Nations (UN) report in 2009, there are approximately 350–500 million Indigenous peoples in 90 countries around the world [3, 4]. The International Labour Organisation (1989) defines Indigenous people as “tribal peoples in independent countries whose social, cultural, and economic conditions distinguish them from other sections of the national community and whose status is regulated wholly or partly by their own customs or traditions or by special laws or regulations; and peoples in independent countries who are regarded as Indigenous because of their descent from the populations who inhabited the country, or a geographical region to which the country belongs, at the time of conquest or colonisation or the establishment of present state boundaries and who, irrespective of their legal status, retain some or all of their own social, economic, cultural, and political institutions [5].” Despite variance in the magnitude of health disparities, globally Indigenous peoples experience a higher burden of disease than their non-Indigenous counterparts [6]. The life expectancy of the Indigenous population is up to 20 years lower than the non-indigenous population and are more vulnerable to the impacts of climate change, natural hazards, and outbreaks such as COVID-19 [7]. The disadvantage, as a consequence of collective histories of oppression and marginalization, has resulted in increased rates of infections and chronic diseases [8]. Continuing impacts of colonialism, community displacement, and land appropriation combined with socioeconomic differences create complex barriers to accessing healthcare for Indigenous peoples [5].

Oral health is not exempt from health disparity trends among Indigenous populations and untreated dental caries, less restored teeth and periodontal disease have been reported to be higher [9–11]. Oral health is multidimensional, including emotional, physical, psychological, and social realms that are vital to general well-being [12, 13]. Oral diseases have serious health and economic ramifications and are among the most widespread diseases globally [9]. Poor oral health among the Indigenous group not only impacts the oral cavity but can result in debilitating pain, limited social interactions, difficulties eating and speaking, embarrassment, and reduced quality of life [10, 14].

According to Global Burden of Disease (GBD) estimates, severe periodontitis is the 11th most prevalent disease in the world [15]. The global prevalence rate for severe periodontal disease is gradually increasing despite efforts to prevent and control the progression of the disease [16]. Gingivitis and periodontitis are the most common oral infections with an overall global prevalence ranging from 20 to 50% [16]. The initial form of periodontal disease, gingivitis, presents as gingival bleeding, clinical inflammation, redness of the gingiva, and pain and if this condition is left untreated it progresses to clinical loss of connective tissue attachment, alveolar bone loss, and subsequently loss of tooth structure [17]. Periodontal disease results from interactions between bacteria and a variety of host risk factors, such as poorly managed diabetes, long-term tobacco smoking, stress, and genetic predisposition [18]. Several researchers have reported that the Indigenous population has a higher burden of periodontal disease than the non-Indigenous population [11, 19, 20] and often experience various barriers in accessing and utilizing dental services [20]. Therefore, it is crucial to identify the global estimates of periodontal disease that has been consistently present and increasing in the Indigenous community. The findings from this review would aim to facilitate researchers, clinicians, policymakers, and Indigenous communities in decision-making approaches to improve periodontal health.

A search of relevant databases showed no current or proposed systematic reviews regarding the prevalence of periodontal disease among Indigenous populations compared with non-Indigenous populations. To our knowledge, this will be the first evidence to highlight the disparities in the occurrence of periodontal disease among Indigenous populations and the first to report a comparative synthesis of results. A preliminary search of MEDLINE, Scopus, and EBSCOhost identified published data that fits within the inclusion criteria for this review. The research question of our review is “what is the magnitude of the disparity in the prevalence of periodontal disease among Indigenous populations compared to non-Indigenous/general populations globally among observational studies?”

## Methods

The proposed systematic review will be conducted following the Joanna Briggs Institute (JBI) methodology for systematic reviews of prevalence [21]. The systematic review protocol has been registered in the International Prospective Register of Systematic Review (PROSPERO), registration number CRD42020188531. For reporting of this systematic review, the Preferred Reporting Items for Systematic reviews and Meta-Analysis (PRISMA) Protocols guidelines (Additional file 1) will be followed [22].

### Inclusion criteria

#### Participants

The review will include studies that have reported the prevalence of periodontal disease among Indigenous populations compared with non-Indigenous populations. This study will include Indigenous peoples from Australia (Aboriginal or Torres Strait Islanders), Canada (Inuit, Metes, First Nations), New Zealand (Maori), USA (American Indian and Alaskan Natives), Brazil (Amerindians, Xingu, Xavante Indians), and other countries, including but not limited to China, South Asia, Southeast Asia, South America, Africa, Central America, Arabia, former Soviet Union, Scandinavia, and the Pacific Islands [23]. Participants of all age groups, including children and adults, will be eligible for inclusion. The inclusion of studies will not be restricted by participant sex or geographical location.

The Indigenous status of participants will be defined by guidelines that are outlined in Article 33 of the UN Declaration on the Rights of Indigenous Peoples (UNDRIP) [24]. As such, Indigeneity identified through self-identification, parent reporting for children, and utilization of country-specific identity registration systems would be eligible for inclusion.

#### Condition

This review will include all papers that have assessed the prevalence of periodontal disease, regardless of the definition of periodontal disease used. The definition of periodontal disease can be based on periodontal probing depth (PPD), clinical attachment level (CAL), or both [25]. Indices that assess periodontal disease would also be included such as community periodontal index (CPI) or periodontal index (PI) [26]. Measures of risk indicators for periodontal disease will be considered as secondary outcomes, such as the prevalence of bleeding on probing (BOP) from the gingiva, prevalence of calculus, and prevalence of plaque [25].

#### Outcomes

The primary outcomes of this review will be as follows:

1. The mean prevalence (%) of periodontitis among Indigenous people compared to non-Indigenous people according to different definitions of periodontitis.
2. The mean number of sites (%) associated with periodontitis.

The secondary outcomes of this review will be as follows:

1. The mean prevalence (%) of plaque index at a factor greater than 2.
2. The mean prevalence (%) of gingival index at a factor greater than 2.
3. The mean prevalence (%) of calculus.
4. The mean prevalence (%) of BOP.

#### Context

Our review will consider studies that report the prevalence of periodontal disease in Indigenous populations from all countries without any geographic restriction. Participants can be recruited from a variety of sites, such as community settings, hospital/ clinical settings, or school settings.

#### Types of studies

This review will consider descriptive observational studies including descriptive cross-sectional studies that have comparative measures of periodontal disease between Indigenous versus non-Indigenous populations. Analytical observational studies will also be included. Data from national surveys, government reports, censuses, and government registries will additionally be considered. Baseline data from clinical trials which report on the prevalence of periodontal disease relevant to our inclusion criteria will be included. If a similar data set is used in more than one study for the same geographical area and periodontal outcome then the primary dataset will be used.

The exclusion criteria for this review will be as follows: (1) analytical or descriptive observational studies without a comparison group; (2) studies that do not measure periodontal disease through clinical assessment or have not reported the periodontal data separately for Indigenous and non-Indigenous populations; (3) studies that assess periodontal parameters through self-oral assessment; (4) studies with non-extractable data; (5) case reports, literature reviews, including systematic reviews and scoping reviews, letters, commentaries, opinion pieces, and editorials.

### Search strategy

For our review, we will employ a three-stage search strategy. Firstly, a limited search of MEDLINE will be conducted to identify articles on this topic. The reviewers will analyze the text words contained in the title and abstract of the relevant articles and the index terms to describe the articles to develop an appropriate search string. The second step will involve using keywords and MeSH terms (Medical Subject Headings) to capture all relevant articles across MEDLINE, Scopus, and EBSCOhost (Dentistry and Oral Sciences Source), University of New Mexico Native Health Database, Bibliography of Native North Americans, and the Australian Indigenous HealthInfoNet. All electronic databases will be searched from review inception to February 2021. Finally, a reference list will be made of all selected articles and free-hand bibliography searching of relevant articles will be done to identify additional studies that might have been overlooked during the electronic search. Narrative reviews and standard textbooks related to the topic will be searched to identify relevant articles. The search strategy for MEDLINE has been described in Appendix 1.

For unpublished data, a similar search will be conducted in Open Grey and ProQuest Dissertation. The authors will conduct an online search using a combination of keywords such as “Indigenous” or “Aboriginal” and “periodontitis” or “periodontal disease” or “gingivitis” or “gingival disease” to find relevant data from national oral health surveys, government reports, national censuses, and government registries. Websites and resources that have comparison data for periodontal disease among the Indigenous and non-Indigenous population will be considered for inclusion. All websites from the search result would be screened by two reviewers for eligibility for inclusion and a table will be made in a spreadsheet recording the title, URL, and date accessed (Appendix 2). We will also contact experts in the field for additional unpublished data and advice on published work (e.g., E Kruger (Australia), R Arantes (Brazil), T Batliner (USA), J Broughton (New Zealand), and H Lawrence (Canada)).

### Reviewer calibration

Calibration will be done before title and abstract screening and the study selection process. Both reviewers (SN & BR) will be calibrated for the screening process on a random sample of 5% of the total number of citations. Both reviewers will screen the selected articles independently and in duplicates. In case of any discrepancy, the outcome will be discussed among the reviewers until a consensus is reached and in case a consensus is not reached a third reviewer (LMJ) will be consulted. A kappa score will be calculated as a screening reliability score among the reviewers.

### Study selection

Findings from the search strategy will be catalogued using EndNote X9 Version 3.3 (Clarivate Analytics, Philadelphia, PA, USA) and duplicate citations will be removed. The study selection process will be carried out in two steps: (1) title and abstract screening, (2) full-text screening.

The abstract and titles will be screened against the inclusion and exclusion criteria. For inclusion, studies must include a comparison of prevalence of any periodontal parameter between Indigenous and non-Indigenous population. The next step will be to retrieve the full text of all potentially relevant articles and import them into the JBI System for the Unified Management, Assessment and Review of Information (JBI SUMARI). The two independent reviewers (SN and BR) will assess the full text in detail for eligibility. Any reason for exclusion will be recorded in JBI SUMARI. The results of the search will be represented in a flow diagram according to the PRISMA guidelines [27].

### Assessment of methodological quality

Eligible studies will be critically appraised by two independent reviewers (SN and BR) at the study selection stage using the standardized critical appraisal instrument in JBI SUMARI for prevalence studies [21, 28]. Any appraisal disagreements will be resolved through mutual discussion or consultation of a third reviewer (LMJ) where necessary. The JBI SUMARI instrument [21] consists of a standardized checklist to ensure consideration of factors such as (1) sampling frame, technique, and size, (2) subject and setting detail, (3) data analysis, (4) methods for identification of the condition, (5) reliability of methods used for measuring the condition, and (6) adequacy of response rate. If any publications have missing information, the authors will be contacted via email. The results of the critical appraisals will be reported in narrative and table form. All studies, regardless of the results of their methodological quality, will undergo data extraction and analysis (where possible).

### Data extraction

The investigators (SN and BR) will perform data extraction from included articles using a customized data extraction form generated in an excel spreadsheet. The extraction tool will be pilot-tested on a random sample (5%) of included studies and revised until a consensus is reached on the key variables and outcomes. Any disagreements that arise between the reviewers will be resolved through discussion, or by discussing with a third reviewer (LMJ). Authors of included papers will be contacted via email to request missing or additional data where required. The following fields will be included (Appendix 3):

1. Study characteristics: study unique reference number, last name of the first author, year and place of publication, study setting, sampling design, sampling frame, and sample size calculations.
2. Participant characteristics: total number of cases, the total number of controls, definition of cases and controls, mean age, method of data collection, parameters of periodontal disease, and periodontitis definition used.
3. Outcome measures: the mean (%) prevalence of periodontitis and mean (%) number of sites involved with periodontitis, mean (%) prevalence of bleeding sites, mean (%) prevalence of sites with calculus, mean (%) prevalence of sites with dental plaque, and mean (%) prevalence of sites with gingival inflammation.
4. Overall findings: the outcome of the study with the main results.

### Data analysis and synthesis

Where appropriate, extracted data will be collated for meta-analysis using the JBI SUMARI software [28]. Where statistical pooling is not possible, findings will be presented in narrative form using tables to aid in data presentation. For this systematic review, Indigenous populations will be considered as the experimental group and non-Indigenous populations as the control group. The mean (%) prevalence and standard deviation (SD) of periodontitis would be the effect estimate from each study. Random effects models will be used for analysis and will be reported as the standardized mean difference with a 95% confidence interval for the differences between the Indigenous and non-Indigenous populations. The random-effects model allows for the differences in the outcome effect from study to study occurring due to differences in the population characteristics (such as age and geographic location) and study characteristics (study sample and sampling design) and other factors [29]. For individual studies, the weight will be calculated on the inverse of the variance. To assess heterogeneity standard chi-squared, Tau squared, and *I*^2^ tests will be used. The *I*^2^ index will be interpreted as low, moderate, or high inconsistency if the values are equal to 25%, 50%, and 75% respectively [30]. To address heterogeneity we will conduct a subgroup meta-analysis and omit studies that have been identified as outliers by visualizing the forest plots. A subgroup analysis of different periodontal disease definitions, age, publication type, and geographical location will be done. Sensitivity analyses will be conducted to test decisions made during the process of conducting the review. Publication bias will be assessed using Egger’s test and visualizing the funnel plot asymmetry [31].

## Discussion

This protocol describes a planned systematic review and meta-analysis to estimate the pooled prevalence of periodontitis among Indigenous and non-Indigenous populations. Previous systematic reviews [11, 32, 33] have shown the prevalence of periodontal disease only among Indigenous populations without any comparison to general populations and has been restricted to specific countries without any global comparison. A comparative analysis is crucial in understanding the magnitude of the diseased condition affecting Indigenous peoples. The prevalence of periodontal disease affecting Indigenous adults is documented as being substantially higher among Indigenous than non-Indigenous groups [11, 34–36], reaching as high as 97.8% [36].

Exploration of the burden of periodontal disease experienced by Indigenous peoples is important due to the continuous nature of the disease and the possible result of bone loss. Identifying variances in the experience of periodontal disease is critical due to the common experience of inflammatory response similar to other systemic conditions [37, 38]. There are established links between periodontal disease and heart and lung disease, diabetes, strokes, and low birth weight of babies of mothers with periodontal disease [39–42]. Periodontal disease is irreversible and successful treatment only restores periodontal health with reduced and compromised periodontium. The anatomical damage from the previous periodontal disease continues to persist, and the inverse architecture of the soft tissues may impair further plaque control. This makes maintenance of periodontally compromised teeth very difficult and eventually leads to loss of tooth structure [43]. If Indigenous populations have a higher burden of periodontal disease, such early tooth loss would impact the overall quality of life and general health and thus increase the economic burden of the country [44]. Findings of this systematic review will be relevant to policymakers and public health officials who inform oral health practices and interventions for Indigenous communities.

### Supplementary Information

**Additional file 1.** PRISMA-P 2015 Checklist.

## Data Availability

The dataset that would be generated and analyzed for the current systematic review will be available from the corresponding author on reasonable request.

## Appendix 1

### Search strategy

Search string for OVID MEDLINE conducted on February 2021:

1. exp Periodontitis.mh
2. noexp Periodontal Diseases.mh
3. noexp Gingival disease*.mh
4. Gingival recession*.mh
5. Gingivitis.mh
6. Gingival pocket*.mh
7. Periodont*.mp
8. Plaque.mp
9. Oral hygiene.mp
10. OR/1-9
11. First nation or First nations.mp
12. Pacific islander or Pacific islanders.mp
13. Torres strait islander or Torres strait islanders.mp
14. Aboringin*.mp
15. Alaska*.mp.
16. Aleut*.mp.
17. Amerind*.mp
18. Arctic.mp
19. Aymara.mp
20. Bushmen.mp
21. Chukchi.mp
22. Chukotka.mp
23. Circumpolar.mp
24. Eskimo*.mp
25. Greenland*.mp
26. hmong.mp
27. indian*.mp
28. indigen*.mp
29. inuit*.mp
30. inupiaq.mp
31. Inupiat.mp
32. Khanty.mp
33. Maori*.mp
34. Mapuche.mp
35. Metis.mp
36. Native*.mp
37. Navajo or Navajo
38. nenets.mp
39. quechua.mp
40. sami.mp
41. samoan.mp
42. Siberia*.mp
43. Skold.mp
44. Tribal.mp
45. Tribe.mp
46. Xingu*.mp
47. Yup’ik.mp
48. zuni.mp
49. African continental ancestry.mp
50. Asian continental ancestry.mp
51. Arctic regions
52. Oceanic ancestry.mp
53. Indigenous health services.mp
54. Ethnic group*.mp
55. OR/11-54
56. 10 AND 55
57. Limit 55 to humans

### Key

Mh = MeSH heading

mp = multipurpose-searches in title, original title, abstract, subject heading, name of substance and registry word fields

*wildcard symbol = broadens the search strategy by capturing the denoted word stem and all other derivatives beginning with the same letters

Exp = exploded indexing terms (MeSH heading)

Noexp = non exploded indexing terms (MeSH headings)

## Appendix 2

Table 1

**Table 1:** Data search for websites

Source Name	URL
Australian Indigenous Health Info Net	http://www.healthinfonet.ecu.edu.au/
Australian Institute of Health and Welfare	https://www.aihw.gov.au/
Indigenous: Australian Government	http://www.indigenous.gov.au/
The Lowitja Institute (Australia’s National Institute for Aboriginal and Torres Strait Islander Health Research)	http://www.lowitja.org.au
The National Oral Health Promotion Clearinghouse	https://www.adelaide.edu.au/arcpoh/oral-health-promotion/
The Brazilian Institute of Geography and Statistics	https://www.ibge.gov.br/en/home-eng.html
Aboriginal Affairs (Government of New Brunswick)	http://www2.gnb.ca/content/gnb/en/departments/aboriginal_ affairs.html
Aboriginal Portal	http://aboriginal.ubc.ca/
Aboriginal Affairs and Northern Development (Government of Canada)	https://www.aadnc-aandc.gc.ca/
Canadian Electronic Library’s Public Documents Collection	http://www.canadianelectroniclibrary.ca/Default.aspx
Canadian Public Health Association	http://www.cpha.ca/en/default.aspx
Circumpolar Health Database	http://www.aina.ucalgary.ca/chbd/
First Nations Health Authority	http://www.fnha.ca/
Ministry of Government Relations (First Nations Metis and Northern Affairs) (Government of Saskatchewan)	https://www.saskatchewan.ca/residents/first-nations-citizens/saskatchewan-first-nations-metis-and-northern-affairs-directory
Nunavut Department of Health	http://www.gov.nu.ca/health
NWT Health and Social Services	http://www.hss.gov.nt.ca/
Statistics Canada	https://www.statcan.gc.ca/eng/start
Yukon Government Department of Health and Social Services	http://www.hss.gov.yk.ca/
Ministry of Health Manatū Hauora	https://www.health.govt.nz
Toi Te Ora Public Health (Government of New Zealand)	https://www.govt.nz/organisations/toi-te-ora-public-health/
Statistics Norway	https://www.ssb.no/en/tannhelse
Alaska Department of Health and Social Services	http://dhss.alaska.gov/dph/wcfh/Pages/oralhealth/report.aspx
Centers for Disease Control and Prevention	https://www.cdc.gov/nchs/fastats/dental.htm
Indian Health Service (Federal Health Program for American Indians and Alaska Natives)	https://www.ihs.gov
Health Info Net	http://www.healthinfonet.ecu.edu.au
OAISter	http://oaister.worldcat.org/
Organisation for Economic Co-operation and Development	https://www.oecd.org

## Appendix 3

Table 2

**Table 2:** Data extraction instrument

Variable	Definition
Study Characteristics	
SID	Unique identification number of study
Author	Last name of first author
Year	Year of publication
Country	Country of study conducted
Study design	Design of the study
Setting	Describes the location of the study as defined by the study authors. For studies that use secondary data, the dataset is described.
Samplemethod	Describe the sampling method
Samplesize	Did the researchers calculate the sample size (yes = 1, no = 2, not clear = 99)
Participant characteristics	
Cases	Describe the Indigenous population
Control	Describe the controls population
Cases_n	Total number of cases
Control_n	Total number of control
age_mean, age_sd	Age of included participants [mean (SD), median (IQR), or categorical age, as reported]
collection_method	Describe the method to collect data.
perio_para	Describe all the periodontal parameters assessed
perio_def	Describe the periodontitis case definition
Outcome measure	
prev_period, mean(%), SD	Prevalence of periodontitis mean % and SD
prevsites_period, mean(%), SD	Prevalence of mean number of sites involved with periodontitis (mean% and SD)
prev_calculus, mean(%), SD	Prevalence of calculus (mean% and SD)
prev_bleeding, mean(%), SD	Prevalence of bleeding (mean% and SD)
prev_plaque, mean(%), SD	Prevalence of plaque score of more than 2 (mean% and SD)
Overall	Overall results of the study.

## Abbreviations

BOP: Bleeding on probing
CAL: Clinical attachment level
CPI: Community periodontal index
JBI SUMARI: Joanna Briggs Institute System for the Unified Management, Assessment, and Review of Information
PI: Periodontal index
PPD: Periodontal probing depth
PRISMA: Preferred Reporting of Items for Systematic Review
UN: United Nations
UNDRIP: UN Declaration on the Rights of Indigenous Peoples
